# From Mechanisms to Meaningful Recovery: Integrating Biology, Technology, and Ethics in Traumatic Brain Injury Care

**DOI:** 10.3390/life16030366

**Published:** 2026-02-24

**Authors:** Seyed Ahmad Naseri Alavi

**Affiliations:** Department of Neurosurgery, Tabriz University of Medical Sciences, Tabriz 5169664339, Iran; dr.arsalan2010@gmail.com

## 1. Introduction

Traumatic brain injuries (TBIs) remain one of the most persistent and complex problems in clinical neuroscience. Even as emergency systems, neurosurgical techniques, and critical care continue to improve, many patients who survive the initial injury are left with long-standing cognitive, visual, emotional, and functional deficits that reshape everyday life. For clinicians, this is the familiar paradox of neurotrauma: survival is only the beginning, and recovery is rarely linear.

Part of the difficulty lies in the nature of the TBI itself. A brain injury is not a single disease; it is a spectrum that involves molecular cascades, structural disconnection, biomechanical forces at the moment of impact, and long-term rehabilitation needs. Meaningful progress therefore depends on connecting these layers rather than studying them in isolation. The contributions assembled in this Special Issue reflect that translational view, spanning biomarkers, imaging, computation, experimental therapeutics, biomechanics, rehabilitation outcomes, and, importantly, the ethical context in which care is delivered [1,2,3,4,5,6,7,8].

## 2. Biological Markers and Precision Prognostication

Early prediction after severe TBIs is one of the major practical challenges clinicians face. Conventional predictors, such as the Glasgow Coma Scale and radiographic findings, provide valuable anchors, but anyone who has followed patients longitudinally knows how often “typical” trajectories fail to apply.

In this paper, Naseri Alavi and colleagues [1] offer a clinically grounded approach by examining sex hormone profiles during the acute phase of severe TBI. They reported associations between serum testosterone in males and progesterone in females with recovery of consciousness, suggesting that endocrine signals that are often treated as background physiology may carry prognostic meaning and may differ by sex [1]. Whether such markers ultimately become part of routine prognostic workflows will depend on validation and integration with other modalities, but this work reinforces a broader point: recovery is not determined by the brain “alone”; it is shaped by whole-body biology [1].

## 3. Imaging Substrates of Cognitive and Visual Dysfunction

Some of the most disruptive consequences of TBI are also the easiest to miss. Standard imaging can appear reassuring when patients struggle with attention, executive function, reading, or visual fatigue symptoms that profoundly affect daily functioning.

Almutairi [3] focuses on a frequently under-emphasized domain: accommodative and oculomotor dysfunction after mild TBI. This review outlines how neurometabolic disturbances and diffuse axonal injury can translate into measurable deficits of accommodation, producing symptoms that patients experience as headaches, blurred near vision, or reading intolerance [3]. This is not merely an ophthalmologic footnote; it is a quality of life issue and a rehabilitation target.

Kamble and colleagues [5] extend the imaging narrative into cognition by linking diffusion tensor imaging findings to executive dysfunction measured through Wisconsin Card Sorting Test variables. Their work supports a clinically intuitive idea with quantitative evidence: microstructural white matter abnormalities, especially in frontal networks, map onto the “real-world” executive difficulties patients report long after injury [5]. Together, these studies point toward a future where imaging is not just diagnostic but functionally informative and rehabilitation-guiding [3,5].

## 4. Artificial Intelligence and Data-Driven Rehabilitation

TBI care now generates an enormous amount of data: demographics, physiologic signals, imaging, laboratory measures, functional scores, and rehabilitation metrics. The opportunity and challenge is turning that complexity into decisions that are both accurate and clinically usable.

Orenuga and colleagues review how artificial intelligence is increasingly being applied across TBI care, from neuroimaging interpretation to prognostic modeling and rehabilitation personalization [2]. The promise here lies not in replacing clinical judgment but rather in supporting it, helping clinicians identify patterns too subtle or multidimensional to parse reliably in real time. At the same time, the authors emphasize practical constraints: generalizability, bias, transparency, and the need for robust prospective validation [2].

## 5. Emerging Therapeutic Frontiers

While acute management remains essential, the field continues to look for therapies that do more than stabilize—therapies that meaningfully alter secondary injury cascades and promote recovery.

Pilipović and colleagues provide a broad view of emerging experimental strategies, including precision medicine, stem cell-based repair, nanomedicine, neuromodulation, brain–machine interfaces, and virtual rehabilitation approaches [4]. What stands out is how these avenues, though diverse, increasingly converge on a shared goal—individualized intervention aligned with patient biology and functional needs—rather than a one-size-fits-all pathway [4].

## 6. Biomechanics and Mechanisms of Injury

Clinical neurotrauma begins with physics. Yet the translation from “force” to an “injury pattern” is rarely straightforward at the bedside.

Zhang and colleagues use finite element modeling to explore blunt head trauma under different strike velocities and bat sizes, quantifying intracranial pressure changes, stress distributions, and injury severity indicators [6]. Studies like this can feel far from the ward, but they matter because understanding thresholds and mechanical signatures informs prevention, forensic interpretation, and ultimately how clinicians conceptualize mechanism-based risk [6].

## 7. Clinical Complexity and Rehabilitation Outcomes

A TBI is not a single event; it is a clinical course that can reveal new complications over time. Chang and colleagues illustrate this through a rare but high-stakes scenario: a traumatic posterior cerebral artery dissecting aneurysm presenting with a subarachnoid hemorrhage that was successfully treated with reconstructive endovascular techniques [7]. Such reports are reminders to maintain diagnostic vigilance for vascular injury after trauma, especially when clinical trajectories do not fit expected patterns [7].

Rehabilitation outcomes are equally central to meaningful recovery. Tay and colleagues examined the recovery from post-traumatic amnesia (PTA) during inpatient rehabilitation and found that the emergence from PTA was strongly associated with a better discharge functional status and long-term global outcomes [8]. These data resonate with everyday clinical experience: structured rehabilitation metrics are not paperwork—they are powerful signals that can guide planning, resources, and family counseling [8].

## 8. Neurotrauma in Context: Civil Unrest, Pellet Injuries, and Medical Neutrality

Traumatic brain injury (TBI) is not limited to accidents or sports. Patterns of head trauma are also shaped by social instability, interpersonal violence, and periods of civil unrest. In these settings, clinicians increasingly encounter penetrating or projectile-related head injuries caused by so-called “less-lethal” weapons, including kinetic impact projectiles and pellet or shotgun-type munitions.

Despite their name, these weapons are far from benign. A growing body of systematic evidence reveals substantial rates of severe injury, permanent disability, and death when they are used for crowd control [9].

During the Iranian uprising and broader unrest, physicians described a noticeable rise in young patients presenting with head, facial, and ocular trauma caused by pellet projectiles. Similar injury patterns particularly devastating craniofacial and eye injuries have been documented in other regions where such munitions are deployed, highlighting the predictable and often life-altering consequences of these mechanisms [9,10].

Yet the burden extends beyond the physical injuries themselves. Outcomes are also shaped by whether clinicians are able to provide care safely and without fear. In some cases, access to treatment has been restricted; healthcare facilities have been subjected to surveillance or intimidation; and physicians who treated injured civilians have faced questioning, detention, or arrest. These pressures erode medical neutrality, delay urgent care, and discourage both patients and providers, ultimately compromising trauma systems at the very moment they are needed most.

At its core, the protection of healthcare workers is not merely a legal or ethical abstraction but a human necessity. When clinicians cannot work freely, patients suffer sometimes quietly, sometimes invisibly. And when physicians are silenced, the suffering often goes undocumented. In such circumstances, the global medical community has a responsibility not only to advance clinical knowledge but also to stand in solidarity with colleagues and patients who cannot safely speak for themselves, to advocate for the protection of medical neutrality, and to ensure that care remains guided by humanity rather than fear [11].

## 9. Looking Forward

The future of TBI research and, critically, the future of patient care rests on integration rather than isolated innovation. Over the past decade, it has become increasingly clear that no single biomarker, imaging modality, or clinical scale can adequately capture the heterogeneity of brain injuries. Instead, progress will depend on combining fluid biomarkers that reflect axonal injury, neuroinflammation, and glial activation with clinical and demographic variables to refine diagnoses, prognostication, and patient stratification [12,13,14]. 

In parallel, advances in neuroimaging are shifting the field away from purely structural assessments toward approaches that are functionally and network informative. Techniques such as diffusion tensor imaging, resting-state functional MRI, and connectome-based analyses have demonstrated that disruptions in large-scale brain networks often underlie persistent cognitive and behavioral symptoms, even when conventional imaging appears unremarkable [15,16,17]. These tools offer a critical bridge between biological injuries and clinical phenotypes.

The growing availability of large, multimodal datasets has also accelerated the use of computational and data-driven methods in TBI research. Machine learning and predictive modeling approaches are increasingly used to integrate biomarkers, imaging, and clinical variables, with the goal of supporting individualized prognostic estimates and clinical decision-making [18,19,20]. While these tools are not substitutes for clinical judgment, they represent an important step toward precision medicine in brain injury care.

Therapeutic priorities are likewise evolving. Beyond acute neuroprotection, there is growing recognition that recovery after TBI is driven by mechanisms of repair, neuroplasticity, and circuit-level reorganization that unfold over months to years [21,22,23]. Experimental and early clinical studies targeting synaptic plasticity, neuromodulation, and activity-dependent recovery highlight the potential to influence long-term outcomes rather than merely limit early damage [24,25].

This shift has direct implications for rehabilitation. Traditional models emphasizing short inpatient stays and early discharge are increasingly misaligned with the prolonged and often nonlinear trajectory of TBI recovery. Evidence supports the need for longitudinal, multidisciplinary rehabilitation strategies that address cognitive, emotional, and social function well beyond the acute phase [26,27,28]. Outcome assessments must similarly evolve, prioritizing meaningful functional recovery and quality of life rather than short-term metrics alone.

At the same time, the field must confront a reality that extends beyond biology and technology: scientific advances only translate into benefits when care can be delivered safely and equitably. Protecting medical neutrality; ensuring access to timely treatment; and safeguarding clinicians and patients particularly in settings of conflict, civil unrest, or political instability are inseparable from efforts to improve outcomes after brain injuries [29,30,31]. When access to care is disrupted or clinicians are constrained from providing treatment, even the most advanced interventions lose relevance. Alongside these broader directions, our team intends to continue along this trajectory by developing new studies grounded in our prior work, while adapting our goals to reflect emerging clinical and scientific needs [11,32,33,34,35,36].

Taken together, the studies included in this Special Issue reflect this broader, integrative vision. They advance mechanistic understanding, refine clinical tools, and highlight system-level challenges that shape real-world outcomes. Collectively, they move the field toward a model of TBI care that is biologically informed, technologically enabled, ethically grounded, and focused on long-term recovery rather than short-term survival.
